# Polysaccharide Based Polymers Produced by Scabby Cankered Cactus Pear (*Opuntia ficus-indica* L.) Infected by *Neofusicoccum batangarum*: Composition, Structure, and Chemico-Physical Properties †

**DOI:** 10.3390/biom12010089

**Published:** 2022-01-06

**Authors:** Gabriella Santagata, Alessio Cimmino, Giovanni Dal Poggetto, Domenico Zannini, Marco Masi, Alessandro Emendato, Giuseppe Surico, Antonio Evidente

**Affiliations:** 1 Istituto per i Polimeri Compositi e Biomateriali, CNR, Via Campi Flegrei 34, Comprensorio “A. Olivetti”, 80078 Pozzuoli (NA), Italy; giovanni.dalpoggetto@ipcb.cnr.it (G.D.P.); domenico.zannini@ipcb.cnr.it (D.Z.); 2 Dipartimento di Scienze Chimiche, Università di Napoli Federico II, Complesso Universitario Monte Sant’Angelo, 80126 Napoli, Italy; marco.masi@unina.it (M.M.); evidente@unina.it (A.E.); 3 Dipartimento di Farmacia, Università di Napoli Federico II, Via D. Montesano 49, 80131 Napoli, Italy; alessandro.emendato@unina.it; 4 Dipartimento di Scienze e Tecnologie Agrarie, Alimentari, Ambientali e Forestali, Università di Firenze, Piazzale delle Cascine 28, 50144 Firenze, Italy; giuseppe.surico@unifi.it

**Keywords:** scabby canker of cactus pear (*Opuntia ficus-indica* L.), exopolysaccharides (EPS), gel, chemical-physical characterization

## Abstract

*Neofusiccocum batangarum* is the causal agent of scabby canker of cactus pear (*Opuntia ficus-indica* L.). The symptoms of this disease are characterized by crusty, perennial cankers, with a leathery, brown halo. Characteristically, a viscous polysaccharide exudate, caking on contact with air, leaks from cankers and forms strips or cerebriform masses on the surface of cactus pear cladodes. When this polysaccharide mass was partial purified, surprisingly, generated a gel. The TLC analysis and the HPLC profile of methyl 2-(polyhydroxyalkyl)-3-(*o*-tolylthiocarbomoyl)-thiazolidine-4*R*-carboxylates obtained from the mixture of monosaccharides produced by acid hydrolysis of the three EPSs examined in this research work [the polysaccharide component of the exudate (EPSC) and the EPSs extracted from asymptomatic (EPSH) and symptomatic (EPSD) cladodes] showed the presence of d-galactose, l-rhamnose, and d-glucose in a 1:1:0.5 ratio in EPSC while d-galactose, l-rhamnose, d-glucose, and d-xylose at the same ratio were observed in EPSH and EPSD. The presence of uronic acid residues in EPSC was also showed by solid state NMR and IR investigation. Furthermore, this manuscript reports the chemical-physical characterization of the gel produced by the infected cactus pear.

## 1. Introduction

The microbial induction of plant diseases, as well as the interaction between the pathogen and the host plant, are complex processes which usually start with the penetration of the pathogen into the host and go on with the production of virulence factors such as wood degradative enzymes and diffusible phytotoxins [1,2,3,4,5] which are responsible for producing the symptoms of disease in the host plant. Once under the attack of a pathogenic microorganism, the plant activates its defense mechanisms and only when the microorganism overcomes all the protectant barriers, either physical or of chemical nature, it establishes a consistent interaction with the plant, leading to a disease.

Quite widespread in nature, many plants are able to exude complex mixtures of bioactive molecules performing a variety of roles. Plant exudates consist of, among others, polysaccharides, lipids, proteins, organic acids and bases, sugars, and amino acids. An exudate produced by many plant species (Euforbiacee, Papaveracee, Moracee, Sapotacee, Composite Asteracee, etc.) is latex, a milky emulsion, usually whitish—rarely yellow, orange, brown, or red [6,7]—composed of small organic compounds suspended in a liquid dispersion medium. Latex acts as the first line of plant defense against herbivores and pathogens, and it also plays a role in environmental stress conditions [6,7,8].

Other types of exudates are resins, gums, and mucilage. Resins are exudates able to preserve the plant against insects and pathogens. Instead, the gums and mucilage play a role in the response to various forms of stress for the plant or plant cells. Moreover, gums are considered to be pathological products as a result of plant injury or due to drought conditions [9,10]. Extracellular polysaccharides (EPSs) are high-molecular-weight sugar-based polymers that are synthesized and secreted by living organisms (plants, animals, algae, bacteria, and fungi) associated with adaptation, survival, and functionalities [11]. In particular, EPSs have been shown to have antioxidant, anti-tumor, and antiviral properties, they also find multiple applications in different fields of human activities.

Among the substances that are involved in the interaction between a plant and its microbial enemies, EPSs may play a variety of roles. As an example, in the case of pathogenic bacteria EPSs are involved in virulence but also in the activation of plant defense responses or in the regulation of QS-dependent genes [12,13].

Moreover, worthy bioactivities of EPSs have also been reported in some fungal diseases: i.e., *Cephalosporium* stripe of winter wheat [14], oak wilt [15], and Dutch elm disease [16]. A possible role of EPSs has also been reported in the chestnut blight disease caused by *Cryphonectria parasitica*. This fungal species produces large amount of pullulan and a minor polysaccharide fraction, which was found to be a galactan, whose structure consisted of the repeat unit: [→6)-β-d-Galf-(1→5)-β-d-Galf-(1→]_n_ [17]. More recently, two main EPSs, identified as a galactan with the known structure [→6)-β-d-Galf-(1→5)-β-d-Galf-(1→5)-β-d-Galf-(1→]_n_ and a complex branched mannan, were found to be produced in vitro by *Phomopsis foeniculi*, a fungal pathogen of fennel. The crude EPSs fraction and galactan and mannan showed phytotoxic effects—i.e., chlorosis, necrosis, and/or wilting on fennel and on two non-host plants, tobacco and tomato [18]. Additionally, fungi involved in grapevine esca disease complex—i.e., *Phaeomoniella chlamydospora*, *Neofusicoccum parvum*, and Brasilian strains of *Lasiodiplodia euphorbicola*, *Lasiodiplodia egyptiacae*, *Lasiodiplodia hormozganensis*, and *Lasiodiplodia pseudotheobromae*—were shown to produce EPSs. Those obtained by *N. parvum* were characterized as a mannan with a backbone consisting of (1→6)-linked mannopyranose units almost all branched at the 2 position, whereby the arms are made up of 2- and/or 3-linked units. When adsorbed by detached grapevine leaves, these EPS induced likewise reddening and withering depending on the tested concentration and time from absorption [19]. The EPSs produced by *P. chlamydospora* were also used to develop a flow cytometry method that unequivocally identifies some of the toxic metabolites. This method was based on the antibodies arisen against by *P. chlamydospora* EPSs by rat immunization [20].

**Figure 1:**
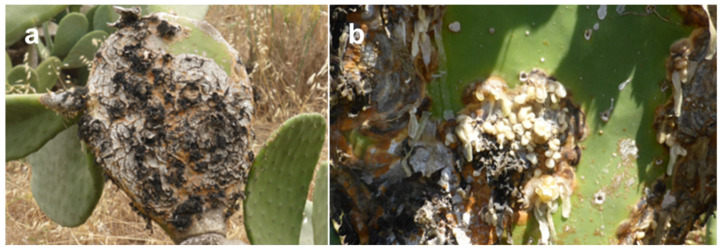
Symptoms of scabby cankers on cladodes of pear cactus (*O. ficus-indica* L.), including concentric, perennial cankers with carbonaceous appearance (**a** panel), and a viscous exudate, caking on contact with air, which leaked from cankers (**b** panel).

An investigation carried out in Lampedusa, the main island of the Pelagie archipelago in the Mediterranean Sea, revealed the presence of a severe disease on cactus pear which was shown to be caused by the fungus *Neofusicoccum batangarum* [21]. The disease symptoms were visible on cladodes and included radially expanding, crusty, concentric, silvery, perennial cankers with a leathery, brown halo. An abundant milky viscous exudate, caking on contact with air, drips from cankers and forms strips or cerebriform masses on the cladode surface. Thereafter, this exudate becomes black in the central part of the canker which takes the aspect of a carbonaceous crust (Figure 1). The exudate was supposed to have a polysaccharidic nature [21].

Polysaccharides from *Opuntia ficus-indica* cladodes have been shown to have several positive effects: for example, skin repairing properties, anti-inflammatory-chondroprotective effects, and several others [22,23]. These EPSs, besides the high molecular weight components, also contain metabolites with different chemical nature and molecular weight distribution. The crude EPSs were used in a wound healing assay to test their cicatrizing capability which induced faster dermal regeneration. The results obtained showed that the whole *Opuntia* mucilage and the low molecular weight components could be proposed as potential active substances in the wound repair [24]. Recently, EPSs of *Opuntia ficus-indica* were bioformuled in a chitosan-based hydrogel film and tested using the same methods. The results showed that the rate of cell migration in EPSs based hydrogels was about 3-fold higher compared to unloaded films, confirming EPSs increased ability to accelerate skin injury repair [25].

The present manuscript reports the isolation, composition, and structural characterization of the polysaccharide component of the viscous exudate (EPSC) on cactus pear (*O. ficus-indica* L.) and its ability to generate a gel. A comparison of the chemical-physical properties of EPSC and EPSs extracted from asymptomatic (EPSH) and symptomatic (EPSD) cladodes is also discussed.

## 2. Materials and Methods 

### 2.1. General Chemica Procedures

One-dimensional solid-state NMR (ss-NMR) experiments were performed on a Bruker^®^ Avance III 400 MHz spectrometer (Karlsruhe, Germany), using a Bruker 3.2-mm magic angle spinning (MAS) wide bore probe head, operating at 400.0 MHz (^1^H) and 100.5 MHz (^13^C), respectively. About 30 mg of powdered samples were packaged into 3.2 mm zirconia rotors and all spectra were recorded at 25 °C, calibrated using KBr [26] and spun at 10 kHz. RF-ramped cross-polarization under magic angle spinning (^13^C CPMAS) and tppm15 high power ^1^H decoupling performed with 90 kHz nutation frequency were applied for ^13^C signal acquisition. Typical acquisition parameters were: 2048 points, 5 s of recycle delay, 35 ms acquisition time, and 8192 scans. The contact time was optimized and set to 1.2 ms in order to achieve an optimal for all carbon signals. ^1^H and ^13^C NMR spectra were recorded at 700 and 175 MHz in D_2_O on a Bruker AVANCE 700 MHz spectrometer (Karlsruhe, Germany), equipped with a cryo-probe, using excitation sculpting water suppression pulse sequence on resonance with the water signals. ESIMS were recorded using ESIMS-TOF system (Agilent 6230B) (Milan, Italy). Analytical TLC were carried out on silica gel (Merck, Darmstadt, Germany, Kieselgel 60, F254, 0.25) plates (Merck,). The spots were visualized by spraying with 10% H_2_SO_4_ in MeOH and heated at 110 °C for 10 min. Gel filtration chromatography was carried out on Sephadex G50 (Sigma-Aldrich, Milan, Italy) column. Standard samples of arabinose, galactose, glucose, mannose, xylose, rhamnose, fucose, and l-cysteine methyl ester and phenyl isothiocyanate were purchased from Sigma-Aldrich and Carlo Erba (Milan, Italy).

### 2.2. Plant Material

The abundant milky viscous exudate, caking on contact with air, which leaked from cankers and formed strips or cerebration masses was collected from infected pear cactus (*O. ficus*-*indica* L.) cladodes infected by *N. batangarum* in Lampedusa island, Sicily, Italy on May 2019. The asymptomatic and symptomatic cladodes were also collected from the same plant and stored at −20 °C until their use. 

### 2.3. Isolation of the Polysaccharide Gel

The fresh milky viscous exudate, and that which caked on contact with air (100 g) were both dissolved in Milli Q water (Merck, Rome, Italy) (400 mL) at room temperature until complete dissolution of the polysaccharide component. The suspension was centrifuged (Sorvall X1 Pro Centrifuge Series, Thermo Fisher Scientific, Waltham, MA, USA) at 7000 rpm at 5 °C for 1 h. The supernatant was dialyzed in 3500 cut-off tube (SpectraPor^®^ 3 Dialysis Membrane, Repligen Corporation Waltham, MA, USA) for 4 days against Milli Q water. A gel separated from the water was collected from the tube content, and lyophilized (Chemistry hybrid pump RC 6, Vacuumbrand, Wertheim, Germany) giving a dried material (9.1 g, EPSC). 100 g of asymptomatic and symptomatic cladodes were minced in a blender (Three-Speed Commercial Blender, Waring^®^, VWR, Radnor, PA, USA) in Milli Q water (400 mL). The suspensions were centrifuged and dialyzed and lyophilized using the same method to obtain 15.9 g (EPSH) and 22.0 g (EPSD) of dried material, respectively.

### 2.4. Acid Hydrolysis of Crude and Purified EPS 

The crude EPSC, EPSH, and EPSD (10.0 mg) were hydrolyzed with 2 M TFA (trifluoroacetic acid) (2 mL) at 120 °C for 2 h using a mini block heater (Greiner Bio-One, VWR, Radnor, PA, USA). The reaction was stopped by evaporation under reduced pressure (Rotovapor Heidolph, Heidolph Instruments GmbH & Co., Schwabach, Germany) by the azeotrope formed adding MilliQ water. The dried residue was dissolved in water and analyzed by TLC on silica gel, eluted with *iso*-PrOH-H_2_O (8:2, *v*/*v*), in comparison with standard samples of arabinose, galactose, glucose, mannose, xylose, rhamnose, and fucose. The same method was used to hydrolyze the fractions EPSC.1-EPSC.3 (2.0 mg) obtained from the Sephadex G50 column (Pharmacia Fine Chemicals Inc., New York, NY, USA).

### 2.5. Gel-Filtration Chromatography 

The crude EPSC (400 mg) was suspended in water and centrifuged at 7000 rpm at 5 °C for 1 h. The supernatant (200 mg) was fractionated by gel permeation chromatography on a column (90 × 2.5 cm) of Sephadex G50 using Milli Q water as eluent and a flow of 30 mL/h. 100 tubes were collected and combined in three main fractions (EPSC.1–EPSC.3) on the basis of their similar TLC profile after spraying with 10% H_2_SO_4_ and heated at 110 °C for 10 min. 

### 2.6. Derivatization of Monosaccharides and the HPLC Analysis of the Aldose Enantiomers Derivative 

d-derivatives: d-glucose (10.0 mg) and l-cysteine methyl ester (12.0 mg) was dissolved in pyridine (300 μL) and heated at 60 °C for 1 h, and then *o*-tolyl isothiocyanate (100 μL) was added to the mixture and heated at 60 °C. After evaporation of the solvent, under reduced pressure, the residue was purified by TLC eluted with CHCl_3_:MeOH:H_2_O (40:10:1, *v*/*v*/*v*) to yield a white amorphous solid (8.0 mg). The same procedure was applied to standards of d-galactose, d-mannose, d-rhamnose, d-fucose, d-arabinose, and d-xylose. The derivatives were analyzed by ^1^H NMR and ESI MS and the spectroscopic data were in agreement with those reported in literatures [27,28]. l-aldose (10 mg) was reacted with the same method described or the d-enantiomers. The mixtures obtained by hydrolysis of EPSC, EPSH, and EPSD (5.0 mg for each sample) were dissolved in pyridine (150 μL) and converted in the corresponding methyl 2-(polyhydroxyalkyl)-3-(*o*-tolylthiocarbomoyl)-thiazolidine-4*R*-carboxylates by reaction with l-cysteine methyl ester hydrochloride (6.0 mg) and phenyl isothiocyanate (50 μL). Analytical HPLC was performed on a Chromaster system (VWR, Hitachi, Darmstadt, Germany) equipped with a RP_18_ Purospher^®^ STAR column (Merck, Darmstadt, Germany) particle size 5 μm, 250 × 4.6 mm) with isocratic elution of 25% CH_3_CN in 0.1% HCOOH solution at a low rate of 0.8 mL/min and the peaks were detected at 250 nm.

### 2.7. Gel Permeation Chromatography

Gel permeation chromatography was performed using a GPC Max Viscotek (Malvern, UK) Phenomenex (Torrance, CA, USA) system equipped with a TDA 305 detector (refractive index, low angle light scattering, right angle light scattering, and viscometer) and UV detector. The column set was composed of a pre-column TSK PWXL and TSK Gel GMPWXL. All the samples were dissolved up a concentration of ≅2 mg/mL and eluted in MilliQ water containing 0.2% NaN_3_ and 0.1 M NaNO_3_. After complete dissolution, samples were filtered through 0.22 μm CA filter. The injection volume was 100 μL, and the flow rate was 0.8 mL/min. The chosen method of analysis was triple point, calibrated with a PEO standard, provided by Viscotek, with a narrow molecular weight distribution. The measurements, performed at 40 °C according to the temperatures of the columns and detectors, ran for 45 min in duplicate.

### 2.8. Attenuated Total Reflection Fourier Transform Infrared Spectroscopy (FTIR-ATR)

ATR-FTIR spectroscopy was carried out on the surface of the EPS powders using a Perkin-Elmer Spectrum 100 (Waltham, MA, USA). The samples were dried under vacuum for 24 h at 60 °C to remove water traces. Spectra were recorded as an average of 16 scans (range: 4000–650 cm^−1^, resolution: 4 cm^−1^). For each sample, a surface mapping of at least three different areas was tested. All the samples were analyzed at room temperature. 

### 2.9. Degree of Methoxylation (DM) Measurement

Degree of methylation (DM) of sample was obtained using a titrimetric method. 200 mg sample was dissolved in 20 mL of MilliQ water and 2 mL of ethanol was added. Two drops of phenolphthalein were added to the mixture and titrated with NaOH solution (0.1 M) until it appeared pale pink. The volume of NaOH was noted as V1. Afterward, the solution was mixed with 10 mL of NaOH and left under stirring for 1 h at room temperature. Then, 10 mL of HCl solution (0.1 M) was added and the mixture was stirred until the pink color disappeared. The solution was titrated again with NaOH solution (V2). The DM value of sample was calculated according to the following equation:

DM (%) = [V2/(V2 + V1)] × 100 (1)

All the measurements were performed in duplicate, and the result is the averaged. value.

### 2.10. Thermogravimetric Analysis (TGA)

Thermogravimetric analysis of EPS based samples residue were performed using a thermogravimetric analyzer TGA/DTG Perkin-Elmer PyrisDiamond, equipped with a gas station. 3–4 mg of samples were placed in an open ceramic crucible and heated from 25 °C to 600 °C at a speed rate of 10 °C/min, under nitrogen flow of 30 mL/min. Before testing, samples were dried at 60 °C under vacuum for 24 h.

### 2.11. Scanning Electron Microscopy (SEM)

Morphological analysis of EPS powdered samples were performed by means of a scanning electron microscope (SEM) (Quanta 200 FEG, 338 FEI, Eindhoven, The Netherlands). Surfaces were coated with a homogeneous layer (18 ± 0.2 nm) of Au and Pd alloy by means of a sputtering device (MED 020, Bal-Tec AG, Tucson, AZ, USA). The micrographs were performed at room temperature, in high vacuum mode. By means of energy dispersive X-ray spectroscopy (EDS), it was possible to perform the chemical analysis of selected microscopic regions. EDS was performed in the SEM by means of an Oxford Inca Energy 250 System equipped with an INCAx-act LN2-free detector, using an accelerating voltage of 20.0 kV.

## 3. Results and Discussion

### 3.1. Isolation and Chemical Characterization of Polysaccharides

The milky viscous exudate collected from pear cactus (*O. ficus-indica* L.) infected by *N. bagantarum* was dissolved in MilliQ water and centrifuged to separate the polysaccharide fraction from the cankered plant part. The viscous solution was dialyzed against a large volume of MilliQwater and surprisingly generated a gel already observable in the dialysis tube. Thus, the gel by lyophilization yielded a crude EPSs (EPSC). The same isolation procedure was applied to obtain the crude EPSs present in the asymptomatic (EPSH) and symptomatic (EPSD) cladodes collected from infected pear cactus. The three polysaccharide samples were hydrolyzed with 2M TFA at 120 °C for 2 h and the monosaccharide residues were analyzed by TLC in comparison with standard samples. The presence of galactose, rhamnose, and glucose was observed in EPSC. More complex mixtures showing the presence of galactose, rhamnose, glucose and xylose were evidenced in both EPSH and EPSD samples. The monosaccharide composition was confirmed by HPLC analyses using the method of Tanaka et al. (2007) [27]. Briefly, the mixtures of monosaccharides obtained by acid hydrolysis of EPSC, EPSH, and EPSD were converted in the corresponding methyl 2-(polyhydroxyalkyl)-3-(*o*-tolylthiocarbomoyl)-thiazolidine-4*R*-carboxylates by reaction with l-cysteine methyl ester hydrochloride and phenyl isothiocyanate. The UV detection at 250 nm of the eluate containing these like derivatives allow to discriminate d- and l-enantiomers of aldoses. The *t*_R_ values of d- and l-aldoses (glucose, galactose, mannose, rhamnose, fucose, xylose, arabinose) after treatment with l-cysteine methyl ester and *o*-tolyl isothiocyanate, were compared with that of same derivatives obtained from the monosaccharide mixture yielded by the acid hydrolysis of the crude three EPSs (EPSC, EPSH, and EPSD) exudates. The analysis of chromatographic profiles allowed to recognize the presence of d-galactose, l-rhamnose, and low d-glucose in 1:1:0.5 ratio in EPSC samples, while d-galactose, l-rhamnose, d-glucose, and d-xylose at the same ratio were observed in EPSH and EPSD samples.

### 3.2. Gel Permeation Chromatography (GPC)

**Figure 2:**
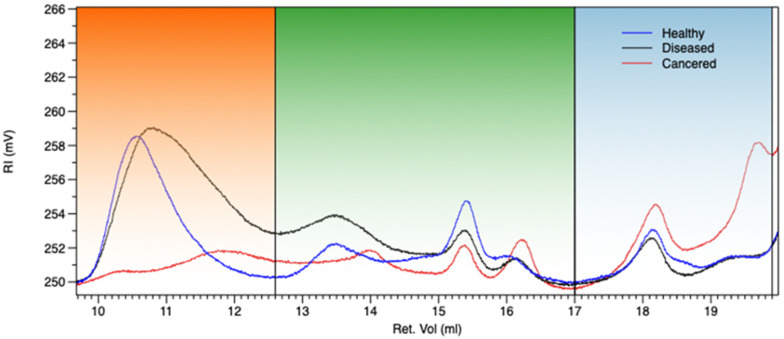
Chromatograms at refractive index detector of EPS based samples: EPSH (blue), EPSD (black), EPSC (red).

Gel permeation chromatography (GPC) allows to evaluate the distribution of polymer molecular weights and to compare likely structural variations among samples [29]. In Figure 2, the chromatograms of EPS based samples are reported while in Table 1, molecular weight parameters, intrinsic viscosity, and weight percentage of the same are detailed. From the superimposed chromatograms, three different Mw regions in retention volume ranges of 9.0–12.6 mL, 12.6–17.0 mL, and 17–19.8 mL can be detected. The first region of chromatograms, regarding the higher polymer molecular weight, evidenced a strong decrease of Mw, Mn, IV, and % weight fraction (%wt.f.) passing from healthy to cankered EPS samples, as specifically detailed in Table 1. This behavior could be ascribed to the fungal activity strongly influencing EPS depolymerization process, consequently highlighted by Mw, Mn, and % weight fraction increasing in the second and third regions of the chromatograms, correlated to the development of oligomers and simple sugars. A significant note regarded the cankered sample, showing the highest value of %wt.f. in the last region, corresponding to the lowest molecular weights (oligomers and simple carbohydrate). Since the main polysaccharide component of the samples, as previously shown, is high methoxylated pectin able to provide to crosslinking process in presence of simple carbohydrates, it is very likely that the strong gel consistency of cankered exudate is due to the development of a three-dimensional network between fungal affected low molecular weight pectin and simple sugars.

### 3.3. Attenuated Total Reflection Fourier Transform Infrared Spectroscopy (FTIR-ATR)

**Figure 3:**
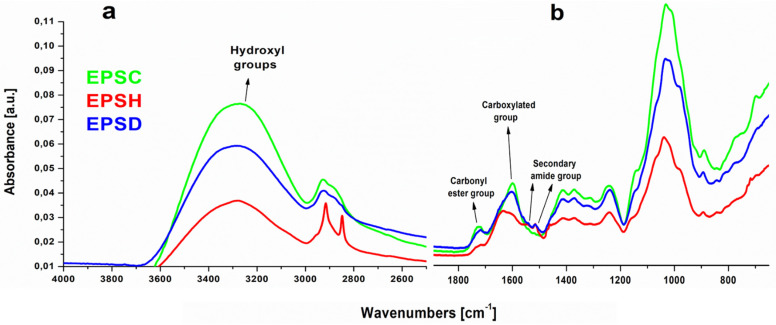
FTIR-ATR spectra of EPS based sample: (**a**) hydroxyl groups engaged in hydrogen bonding, (**b**) carbonyl and fingerprint regions.

Fourier transform infrared spectroscopy is a useful tool in monitoring the main functional groups of polymers. In Figure 3, the spectra of EPS samples have been reported. The spectra showed broad, strong, and superimposed bands in the range 3600–3000 cm^−1^ due to the stretching vibration of O-H groups engaged in intramolecular or intermolecular hydrogen bonding. Based on the previous HPLC results, the main monosaccharides found in EPS-based samples were galactose, glucose, and rhamnose. Since, generally, polysaccharides contain a significant number of hydroxyl groups which exhibit a broad absorption band around that vibrational frequency range, it was in all likelihood confirmed that EPSs polymers were mainly polysaccharides. Moreover, the peaks in the range of 2920–2850 cm^−1^ were associated to the asymmetric and symmetric C-H stretching modes of methyl or methylene groups, usually present in hexoses like glucose or galactose, or deoxyhexoses like rhamnose or fucose [30]. Significantly, the absorption bands around 1720 cm^−1^ were assigned to the stretching vibration of esterified carbonyl groups C=O resulting from uronic acids, previously also detected by NMR analysis (see below) and likely associated with the presence of pectins, as reported by Madera-Santana et al. (2018) [31] for cladodes of *Opuntia spinulifera* plant. This outcome was confirmed by the presence of an additional peak at 1244 cm^−1^ [32]. The region between 1650–1600 cm^−1^ was attributed to the presence of carboxylated groups (COO^-^) associated with the broad and overlapped band at 1403 cm^−1^, respectively attributed to the asymmetric and symmetric stretching vibration of carboxylate ions (COO^−^), widely found in polysaccharide-based structure. Nevertheless, a high pectin esterification degree (about 74%) was found that should be evidenced by higher peak intensity if compared to the carboxylated ones. FTIR-ATR spectra always showed higher intensity of carboxylated group absorption peaks; actually, in the range between 1650–1600 cm^−1^, carbonyl amide stretching vibration groups could also be found. This outcome was also supported by the presence of additional peaks at both 1540 cm^−1^ and 1517 cm^−1^, likely assigned to the NH deformation of amine group and secondary amide group deformation, respectively [33]. On the other hand, it is worth underlining that these secondary peaks, well observable in EPSH and EPSS samples, were not detectable in EPSC sample, thus suggesting the strong reduction of amino acid components in the cankered form of EPS. Actually, it is widely accepted that phytopathogenic fungi express high amounts of hydrolytic and oxidative enzymes, such as proteases and glycoside hydrolases, able to break the peptide bonds in proteins, resulting in smaller peptides and/or amino acids. It is also reported that, in the presence of plant extract, the proteases secretion by filamentous fungi increases [34]. Thus, it could occur that, in presence of *N. batangarum* fungus, the amide components of EPSC exudate strongly decreased thus confirming the FTIR-ATR outcomes. Besides the strong asymmetrical stretching around 1600 cm^–1^, the carbohydrate groups showed another weak symmetric stretching band around 1415 cm^–1^. This was followed by intense absorption patterns between 1300 and 800 cm^–1^. These are collectively referred to as the ‘finger print’ region for carbohydrates because it is unique to a compound and, therefore, allows the identification of major chemical groups in polysaccharides [35]. In this region, the strong vibrational modes corresponding to C-O-C, C-OH and C-C bonds identified the carbohydrate ring. The high intensity peak at around 1030 cm^–1^ indicated that the EPS sample contained pyranose, while the peaks at 982 cm^–1^ and 890 cm^–1^ referred to the absorption of d-glucopyranosyl and α-d-mannopyranose, respectively. Thus, since the presence of the above found vibrational modes in the low frequencies region of the FTIR spectrum, it can be concluded that this range effectively represented the polysaccharide characteristics of EPS samples [36].

### 3.4. Scanning Electron Microscopy (SEM)

**Figure 4:**
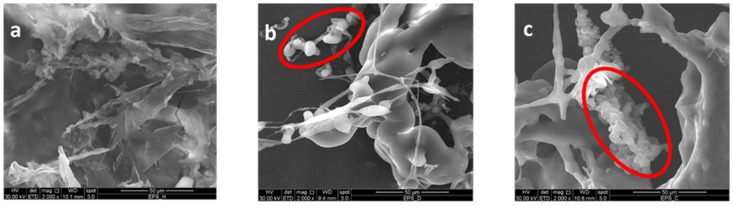
SEM micrographs of EPSH (**a**), EPSD (**b**), and EPSC (**c**) samples. Bar scale for all pictures is 50 µm.

The SEM micrographs of EPS surface-based samples are reported in Figure 4a–c. From the analysis of the pictures of the same magnifications, it is possible to observe a significant difference between healthy and scabby/cankered morphologies. In particular, EPSH sample showed a sheet-like structure with fairly smooth, continuous, and compact surface. This outcome confirmed the previous GPC findings related to the EPSH high molecular weight, able to provide a film-like structure, thus suggesting its potential application as film-forming material [37]. Vice versa, EPSD and EPSC samples evidenced irregular, rough surface, characterized by globular and compressed clusters with interconnected intercellular spaces, typical of compact three-dimensional networks. Actually, the presence of small ovaloid particles well interconnected each other could be due to the formation of gelled structures (see red circles in Figure 4b,c) among pectin and oligosaccharides or simple carbohydrates [38]. This result was particularly evidenced in EPSC sample, in this way supporting the previous hypothesis about the development of crosslinked structures (gel) in this depolymerized sample. 

### 3.5. Thermogravimetric Analysis (TGA) 

**Figure 5:**
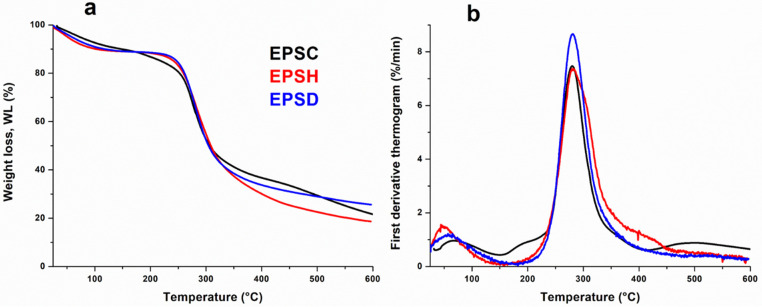
EPS-based sample thermograms: Weight loss (%) (TGA) (**a**) and thermal degradation rate (DTG) (**b**).

Thermogravimetric analysis (TGA) and differential thermogravimetric (DTG) curves of EPS based samples are shown in Figure 5. All curves were normalized with respect to the starting sample size. The thermograms were quite similar and it was possible to detect three different regions of weight loss. The first one, from room temperature up to 150 °C, was attributed to moisture evaporation; in this region, the samples lost about 10% of their water content and, from DTG thermograms accounting for the kinetics of weight loss, it was possible to highlight that EPSH sample evidenced a faster water releasing; the second mass loss process was observed in the range of 150–400 °C and corresponded to the major stage of the thermal weight loss. This region was characterized by the decomposition of the polymer fractions of healthy, diseased, and cankered EPS samples, which resulted in a single sharp DTG peak with a weight loss of about 60%. Actually, it deserves mentioning that EPSH sample showed a slight broadening of the degradation kinetics (see Figure 5b). This outcome could be likely due to the complex decomposition pattern of both polysaccharide and amide fractions of the healthy gel, confirming the FTIR findings. As concerning EPSC sample, it is worth underlining the presence of DTG peaks around 200 °C and 500 °C, likely due to the decomposition of hemicellulose and lignin fraction included inside the exudate gel. Actually, the lignification or suberification process could be a plant reaction to the fungus attack, as evidenced by the browning of the plant and derived exudate gel [39].

### 3.6. NMR Spectroscopic Characterization of the Milky Exudate (EPSC)

**Figure 6:**
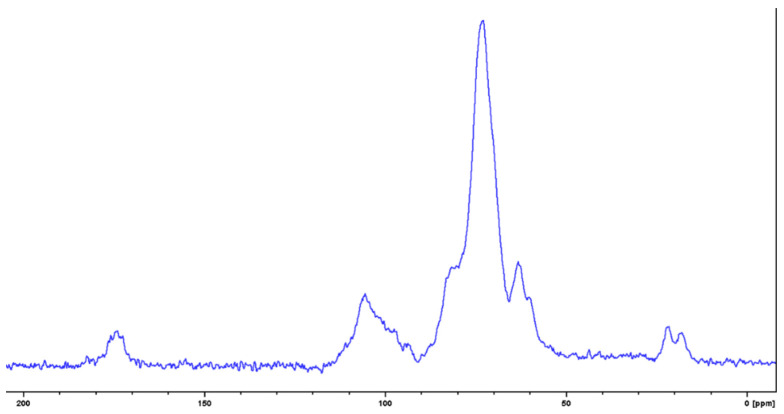
^13^C CP-MAS ssNMR spectrum of EPSC recorded at 400 MHz.

The crude EPSC was analyzed by ^13^C solid state NMR spectroscopy. The spectrum (Figure 6) showed the signals in the region of the anomeric and of the hydroxylated carbons in the range of δ 115.0–95.0 and 82.0–60.0 ppm, respectively. The presence of signals at δ 17.0–22.0 ppm due to methyl group of deoxy sugars and the significant presence of a carboxylic group—probably due to 6-carboxy residues in the range of δ 170.0–179.0—were also observed [40,41], thus confirming the previous FTIR-ATR findings.

The crude EPSC was fractionated by gel-permeation chromatography on a column of Sephadex G-50. Three fractions were obtained (EPSC.1-EPSC.3), hydrolyzed and analyzed by TLC and HPLC using the method above reported [27]. The presence of d-galactose and l-rhamnose was observed in the main fraction (EPSC.2) while only d-glucose was evidenced in the EPSC.3. 

**Figure 7:**
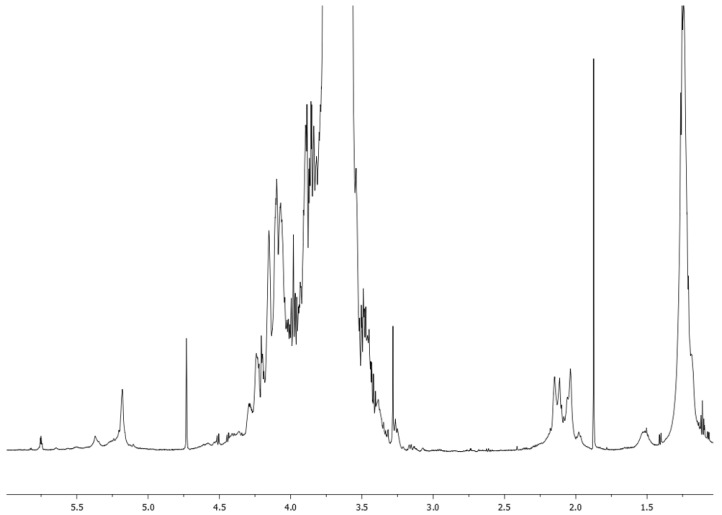
^1^H NMR spectrum of EPSC.2, recorded at 700 MHz in D_2_O.

The fraction EPSC.2 was analyzed by ^1^H NMR recorded in D_2_O solution (Figure 7) and showed the presence of typical signals for anomeric protons at δ 5.4–4.6 ppm, complex signals in the range of δ 4.3–3.2 ppm typical of hydroxylated ring protons and singlets at δ 1.2 ppm for 6-deoxy residues [42,43]. 

**Figure 8:**
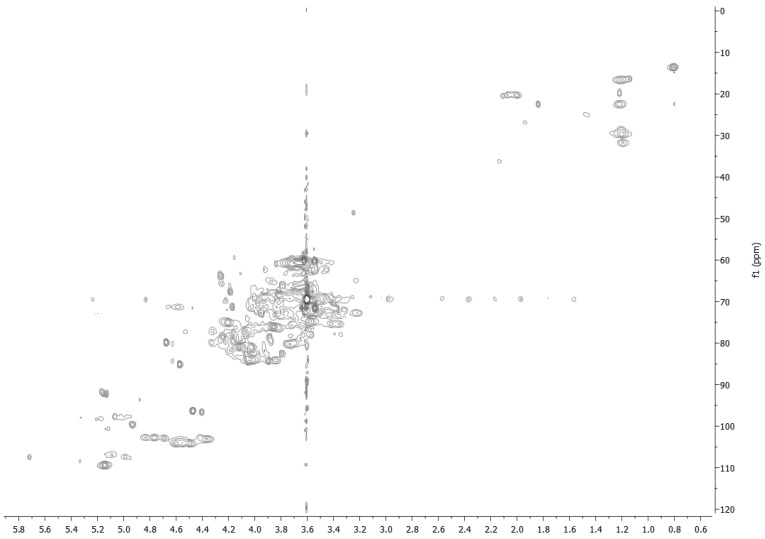
2D HSQC NMR spectrum of EPSC.2, recorded at 700 MHz in ^2^H_2_O.

2D HSQC spectrum of fraction EPSC.2 (Figure 8) confirmed the results obtained by ^13^C NMR in solid state recorded on EPSC and ^1^H NMR in D_2_O solution. In fact, signals at δ 110–95 ppm and δ 85.0–65.0 ppm typical of anomeric and hydroxylated carbons and signals at δ 15.0–30.09 ppm of 6-deoxy carbons were observed [44,45]. However, the spectrum showed the presence of many anomeric and ring carbon signals, suggesting the structure of a branched polysaccharides or of a complex mixture and that it needs further purification in order to determine its structure.

## 4. Conclusions

In conclusion, the polysaccharides come out from the cladodes infected with *N. batangarum* seems structurally different from EPSs isolated from asymptomatic and symptomatic cladodes, and they may represent a simple pathological product of the lesions caused by the fungus on cladodes, just like resins in other plant species. In alternative, they may be part of a defense reaction of the plant aimed at incorporating the pathogen in a viscous material to limit its spread or even its viability if it also possesses antibiotic properties. However, other analyses are needed to clarify these aspects and the deepening of these worthy preliminary results will be the object of a future dedicated paper. Last but not least, further investigations related to the potential applications of EPSC, EPSH, and EPSD samples, as reported above for the EPS extracted by *O. ficus-indica*, are currently in progress and will be detailed in a forthcoming paper.

## Figures and Tables

**Table 1:** Molecular weight parameters (Mw, Mn), intrinsic viscosity (IV expressed as dL/g), and weight percentage fraction (%Wt) of EPS based samples.

Sample	Mw(1) 9.0–12.6 (MDa)	Mn(1) 9.0–12.6 (MDa)	Mw(2) 12.6–17.0 (kDa)	Mn(2) 12.6–17.0 (kDa)	Mw(3) 17–19.8 (kDa)	Mn(3) 17–19.8 (kDa)	IV (1)	IV (2)	IV (3)	%Wt (1)	%Wt (2)	%Wt (3)
EPSH	4.3	1.4	35.5	24.9	2.5	2.3	4.91	0.19	0.05	81	12	7.3
EPSD	2.8	1.3	113	26	1.4	1.2	4.89	0.28	0.08	47.0	38.7	14.2
EPSC	2.9	2.6	103	62	7.6	7.2	3.4	0.04	0.01	54.4	17.6	28.0

## Data Availability

Not applicable.
